# Somatostatin Effect on Growth Factors in Hepatocellular Carcinoma

**DOI:** 10.3390/cimb48020134

**Published:** 2026-01-26

**Authors:** Angeliki Tsakou, George Notas, Costantinos Xidakis, Ioannis Tsomidis, Elias Kouroumalis, Argyro Voumvouraki

**Affiliations:** 1 1st Department of Internal Medicine, AHEPA University Hospital, 54621 Thessaloniki, Macedonia, Greece; angtsak207@gmail.com; 2 Laboratory of Gastroenterology and Hepatology, School of Medicine, University of Crete, Voutes Campus, 70013 Heraklion, Crete, Greece; costas.xidakis@gmail.com (C.X.);; 3 Laboratory of Experimental Endocrinology, School of Medicine, University of Crete, Voutes Campus, 70013 Heraklion, Crete, Greece; 4 Department of Gastroenterology, University General Hospital of Heraklion (PAGNH) School of Medicine, University of Crete, 71500 Heraklion, Crete, Greece

**Keywords:** hepatocellular carcinoma, IGF-1, gastrin, HGF, SCF, VEGF, trophic factors, somatostatin, octreotide

## Abstract

Growth factors play a significant role in the immunopathogenesis of liver diseases, especially liver cirrhosis and hepatocellular carcinoma (HCC). The somatostatin analog octreotide has been used as treatment in advanced HCC, based on its anti-neoplastic effects in vitro. Therefore, the effect of somatostatin and octreotide was studied on several growth factors in patients with HCC. Nineteen patients with advanced HCC were treated with octreotide and compared with thirty-seven patients with viral cirrhosis (19 decompensated) treated with intravenous somatostatin for severe bleeding from portal gastropathy. Five growth factors, namely Gastrin, Insulin-like growth factor 1 (IGF 1), Hepatocyte growth factor (HGF), Stem cell factor (SCF) and Vascular endothelial growth factor (VEGF) were measured in serum before and after treatment with specific commercially available ELISAs. Seventeen healthy individuals and nineteen patients with chronic viral hepatitis C (CAH) were used as pre-treatment controls. Eighteen patients with advanced Primary Biliary Cholangitis (stage III and IV) before and after Ursodeoxycholic acid (UDCA) treatment were also studied. Pre-treatment levels of Gastrin were significantly increased in HCC, cirrhosis and PBC but not in CAH. Levels were significantly reduced by octreotide or somatostatin but also by UDCA in PBC. By contrast, IGF1 showed a mirror image being significantly reduced in HCC, cirrhosis and PBC, but not in CAH. Post-treatment levels were reduced in all groups, but not in PBC. Levels of HGF were significantly increased in HCC and cirrhosis but not in CAH and PBC. They were further increased in HCC after treatment. SCF increased only in HCC and was reduced after octreotide but not after somatostatin treatment. VEGF was reduced in cirrhosis and CAH but not in PBC. It was not significantly increased in HCC, but it was reduced by octreotide and was increased after UDCA. In this retrospective observational study, somatostatin and its analog octreotide have a significant effect on several growth factors involved in HCC pathogenesis.

## 1. Introduction

Several growth factors such as hepatocyte growth factor (HGF), vascular endothelial growth factor (VEGF) and epidermal growth factor (EGFR) participate in liver regeneration after acute liver injury. However, in chronic liver injury, a dysregulation of regeneration in association with the activation of oncogenes leads to uncontrolled cell proliferation and development of hepatocellular carcinoma (HCC). Growth factors are also implicated in oncogenesis by promoting fibrogenesis or angiogenesis. Furthermore, a growth factor with anti-fibrotic effects such as stem cell factor (SCF) may also be involved in HCC pathogenesis [1,2].

Gastrin, a peptide hormone secreted from antral Gastrin-expressing endocrine cells (G cells), has been shown to suppress gastric carcinogenesis [3]. However, the expression of cholecystokinin 2 receptor (CCKBR or Gastrin receptor) has been reported in liver cancer [4]. This receptor is over-expressed in mouse and human HCC but not in the normal liver [5]. Pharmacologic inhibition of the CCK-BR with the receptor antagonist proglumide or genetic deletion of the CCK-BR showed a decrease in genes promoting cell proliferation and oncogenesis and an increase in tumor suppressor genes [6]. The balance between Gastrin and somatostatin regulates hydrochloric acid secretion in the stomach [7].

IGF1 is involved in the regulation of cell growth, but in addition to cell growth IGF1 is an anti-apoptotic factor promoting the survival of tumor cells [8]. Several studies have demonstrated an association between circulating IGF1 levels and increased risk for the development and progression of various cancers, including liver cancer [9,10]. Alterations of the expression pattern of GH-IGF axis were demonstrated in HCC, indicating that this system may be involved in the development of HCC [11]. Moreover, an autocrine production by HCC cells could lead to local IGF1 bioavailability in patients with HCC, leading to tumor progression [12].

There is substantial evidence that VEGF is strongly implicated in the progression of liver cancer. HCC is a hypervascular tumor and several angiogenic factors are overexpressed in tumor cells and the tumor microenvironment [13]. Clinical studies with small numbers of patients suggested that VEGF expression in HCC is associated with advanced disease and reduced median survival [14]. In HCC microenvironment, Kupffer cells and monocyte-derived macrophages are differentiated into tumor associated macrophages (TAMs) that secrete vasoactive molecules and promote tumor progression [15,16]. TAMs activate the STAT3 signaling pathway to stimulate VEGF expression in HCC cells [17].

HGF is produced by hepatic stellate cells and acts on its receptor c-Met, which is localized on the surface of hepatocytes. HGF activates a signal transduction pathway including effector molecules such as PI3K-AKT, which increase DNA synthesis and cell cycle progression during regeneration. Under normal conditions, the HGF/c-Met signaling pathway is tightly controlled [18,19]. Signaling pathways related to c-Met are aberrant in HCC, leading to increased HCC cell proliferation and invasion. Therefore, aberrant expression of HGF is associated with tumor promotion. C-Met mRNA levels are significantly elevated in HCC tissues [20,21]. The HGF/c-Met was also used as a biomarker in HCC diagnosis and prognosis and as a therapeutic target for HCC. It is also implicated in drug resistance during HCC treatment [22]. Details on the implication of HGF/c-Met axis in HCC have been published [23].

SCF is a multi-potent growth factor acting on its receptor c-kit encoded by the KIT gene [24]. Approximately 20% of hepatocytes at the periportal and pericentral regions express c-kit that participates in liver regeneration. Oval cells are the progenitor cells of the liver with a strong expression of c-kit. They can differentiate into various liver cells in specific liver injuries. Transplantation of c-kit positive oval cells improves liver regeneration [25]. The SCF/c-kit signal transduction system is significantly involved in the activation and proliferation of oval cells. In the liver, the malignant transformation of c-kit+ oval cells might be one of the possible mechanisms of HCC development. Oval cells work throughout the development of HCC [26]. On the other hand, overexpression of c-kit is also associated with angiogenesis, which is an additional factor connecting the SCF/c-kit pathway with HCC [26,27,28].

Somatostatin (SST) is a peptide hormone produced by different cells of the body, but mainly by the D cells of the gastrointestinal tract and pancreas. It is generally an inhibitor of several hormones, including growth factors. SST is the ligand of five receptors (SSTR 1–5) with various expressions in different organs [29]. Human hepatocellular cells express SST receptors and their proliferation is inhibited by the somatostatin synthetic analog octreotide [30]. There is substantial experimental evidence that SST may inhibit HCC and other tumors [31]. However, data from human studies are limited. Therefore, we chose five of the better studied growth factors related to carcinogenesis to examine the basic levels of these factors in the different stages of chronic HCV (Chronic hepatitis—CAH, compensated cirrhosis, decompensated cirrhosis, HCV-related hepatocellular carcinoma) compared to normal controls and a non-viral cholestatic liver disease such as PBC. Moreover, the effects of somatostatin or its analog octreotide in cirrhotic patients before and after the development of HCC were studied. Demonstration of a possible additional justification for using these compounds in HCC future trials was a secondary end point.

## 2. Patients and Methods

### 2.1. Study Design

This was a retrospective clinical–laboratory cohort analysis. Serum samples were retrieved from the database of the Laboratory of Gastroenterology and Hepatology, School of Medicine, University of Crete. The database contained anonymized diagnostic information and biochemical and hematological data derived from previous clinical studies.

#### 2.1.1. Patients

Ninety-three patients were included in the study and were compared with seventeen healthy controls selected from the departmental staff to match the age of the patients.

Nineteen patients with advanced, inoperable HCC (age 45–68 years, male 14) and cirrhosis who received octreotide (250 μg t.i.d. octreotide for 4 days followed by 30 mg IM of long-acting octreotide every month) during an earlier study were included. All patients had HCV-related HCC [32].

Nineteen patients with HCV-related decompensated cirrhosis (age 41–70 years, male 12) and eighteen patients with HCV-related compensated cirrhosis (age 37–59, male 15) who received somatostatin for portal hypertensive gastropathy bleeding (an initial injection of 250 μg bolus somatostatin was followed by a continuous infusion of 250 μg/h for 4 days) [33], were also included in the study population. Additionally, 19 patients with chronic viral hepatitis C (CAH, age 36–60 years, male 13) and 18 patients (age 35–63, male 3) with stage III-IV primary biliary cholangitis according to Ludwig [34] and treated with ursodeoxycholic acid (UDCA) were selected.

Decompensated cirrhosis was defined as the presence or history of any ascites, variceal bleeding, hepatic encephalopathy, or jaundice due to disease progression [35]. Bleeding from portal gastropathy alone was not considered as decompensation.

HCC patients were Child–Pugh score B and Barcelona stage C. Patients with compensated cirrhosis were all Child–Pugh score B, while 9/19 patients with decompensated cirrhosis were Child–Pugh C. All HCC and cirrhotic patients had evidence of portal hypertension as judged by the presence of esophageal varices and/or portal gastropathy. Seven of the nineteen patients with decompensated cirrhosis had an episode of variceal bleeding in the past treated by somatostatin infusion and 10/19 were receiving diuretics as required for ascites controls. As mentioned before, the study was not a clinical trial but an effort to compare basic levels of growth factors in different stages of chronic HCV and study the effects of somatostatin and octreotide in HCC and cirrhosis. Therefore, patients were not matched for inflammation or liver reserve.

The diagnosis of PBC was confirmed according to the European Association for the Study of the Liver (EASL) guidelines for PBC [36]. All PBC patients were AMA positive by immunofluorescence, anti-M2 positive by ELISA, and had increased IgM levels and a compatible liver biopsy. They were also sp100 and gp210 positive. They were included in order to identify the basic levels of the growth factors in a model of chronic advanced cholestatic disease.

Diagnosis in all patients was verified by liver biopsy with the exception of those with decompensated cirrhosis. All patients and healthy controls gave informed consent in writing.

The demographics of patients are presented in Table 1.

Study exclusion criteria were as follows: Previous systemic anti-cancer or anti-fibrotic therapies potentially affecting growth factor levels. Any indication of metastatic disease or portal vein thrombosis. Patients under 18 years old. Active infections, autoimmune conditions, or other comorbidities known to interfere with the studied biomarkers. Insufficient or degraded serum samples. Absence of prior informed consent for participation in the study.

Blood was collected from somatostatin or octreotide treated patients before and on the 5th day after drug administration. Blood was collected before and after 6 months of UDCA treatment (13–15 mg/kg body weight) in PBC patients. Serum samples were separated after blood clotting by centrifugation at 1100 g for 10 min and stored at −80 °C until measured.

The research protocol was approved by the Ethics Committee of the University Hospital (23753/2025 and 836/2025). Approval was granted for using database and serum for this particular study. The study was performed in accordance with the principles of the Declaration of Helsinki.

#### 2.1.2. Methods

IGF-1, HGF, VEGF and SCF were measured using the Human Quantikine ELISA kits (R&D Systems, Minneapolis, MN, USA). ELISA for Gastrin I was from Invitrogen (Waltham, MA, USA). All measurements were performed according to manufacturer instructions and read in a BioTekek ELx800 microplate Reader (Winooski, VT, USA).

### 2.2. Statistical Analysis

Results are presented as means ± SD. Box and whiskers plots were used to demonstrate growth factors levels indicating the first quartile, mean, third quartile, the inter-quartile range and outliers. Results before and after treatment are presented as bars ± SD indicating the difference between the pro and post-treatment value.

#### 2.2.1. Comparison Analysis (Baseline Group Comparisons, Before Treatment Administration)

The normality of the data distribution was assessed for each study group using the Shapiro–Wilk test.

For normally distributed data with homogeneous variances, between-group comparisons were performed using one-way ANOVA followed by post hoc analysis with Bonferroni correction. When the assumption of homogeneity of variances was violated, Welch’s ANOVA was applied. For non-normally distributed data, non-parametric tests were used, including the Kruskal–Wallis test for overall between-group comparisons and Dunn’s post hoc test for pairwise analyses. A *p*-value < 0.05 was considered statistically significant.

#### 2.2.2. Paired Analysis

To evaluate treatment effects within each group, pre- and post-treatment values of each growth factor were compared. For normally distributed data, the paired Student’s t-test was applied, whereas for non-normally distributed data, the Wilcoxon signed-rank test was used. Finally, a comparison of the pre- to post-treatment differences was performed for each growth factor using one-way ANOVA with Bonferroni post hoc correction for normally distributed data, or the Kruskal–Wallis test with Dunn’s post hoc comparisons for non-normally distributed data.

To account for multiple comparisons across biomarkers and groups, post hoc adjustments (Bonferroni or Dunn’s correction) were applied where applicable, in order to control for inflation of Type I errors.

The significance level was set at α = 0.05. Exact *p*-values are reported where applicable. When the *p* value was calculated as *p* = 0.000, it was reported as *p* < 0.001 according to standard statistical practice. Effect sizes were calculated to estimate the magnitude and clinical relevance of observed effects, independent of sample size. Specifically, Cohen’s d was used for parametric comparisons, while rank-based effect size measures (r_rb or rank-biserial correlation) were reported for non-parametric analyses. Where applicable, 95% confidence intervals were calculated for parametric estimates.

Given the retrospective nature of the study and the limited sample size in certain subgroups, no a priori power calculation was performed. Τherefore, the potential risk of Type II error, particularly for non-significant findings, is acknowledged and considered in the interpretation of results.

## 3. Results

### 3.1. Comparison Analysis

#### 3.1.1. Gastrin I

**Figure 1:**
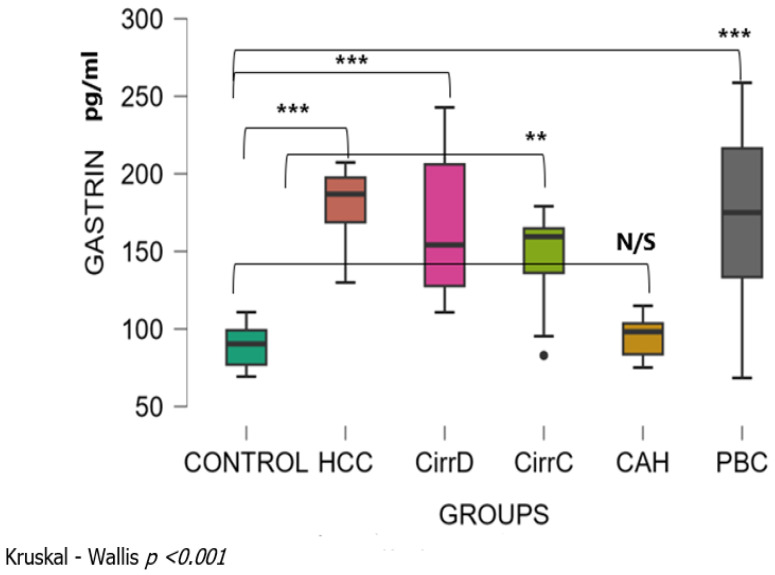
Serum concentrations of Gastrin in the study groups before treatment. The number of cases is given in the patient subsection and is similar in all subsequent figures. CirrD: Decompensated cirrhosis; CirrC: Compensated cirrhosis; CAH: Chronic viral hepatitis C. **: *p* < 0.01, ***: *p* < 0.001.

There was a significant difference among patient groups (Kruskal–Wallis *p* < 0.001). The mean levels of serum Gastrin were 89 ± 13 SD pg/mL for the control group. Levels were significantly increased with a large effect size in HCC patients (179 ± 24, *p* < 0.001, r_rb = 1), in patients with late stages PBC (171 ± 57, *p* < 0.001, r_rb = 0.77) and in patients with decompensated or compensated cirrhosis (168.8 ± 45.3, *p* < 0.001, r_rb = 0.99 and 147 ± 27, *p* < 0.001, r_rb = 0.88, respectively). Values were not different between chronic hepatitis (95 ± 12, *p* = 0.60, r_rb = 0.2) and controls (Figure 1). Dunn’s post hoc test for pairwise analysis showed that there was no significant difference between the groups, with the exception of CAH which was significantly different from all other groups.

#### 3.1.2. IGF-1

**Figure 2:**
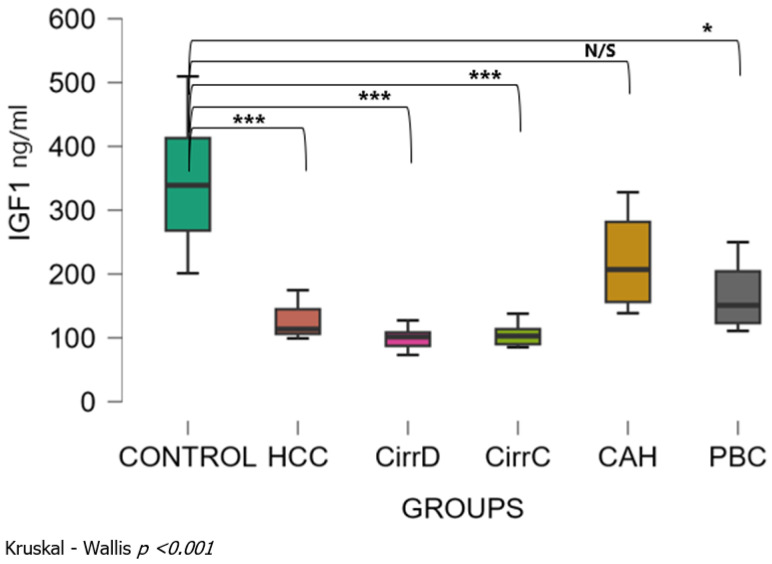
Serum concentrations of IGF1 in the study groups before treatment. CirrD: Decompensated cirrhosis; CirrC: Compensated cirrhosis; CAH: Chronic viral hepatitis C. *: *p* < 0.05, ***: *p* < 0.001.

There was also a significant difference among patient groups (Kruskal–Wallis *p* < 0.001). The mean levels of serum IGF-1 were 343 ± 96 SD ng/mL for the control group. Levels were significantly decreased with a large effect size in HCC patients (124 ± 25, *p* < 0.001, r_rb = 1), in patients with late stages PBC (165 ± 47, *p* < 0.05, r_rb = 0.92) and in patients with decompensated or compensated cirrhosis (99 ± 15, *p* < 0.001, r_rb = 1 and 104 ± 16, *p* < 0.001, r_rb =1, respectively). Values were not different between chronic hepatitis (220 ± 67, *p* = 0.9, r_rb = 0.6) and controls (Figure 2). Dunn’s post hoc test for pairwise analysis showed that there was a significant difference between HCC and CAH and between patients with cirrhosis (both decompensated and compensated) and CAH or PBC.

#### 3.1.3. HGF

**Figure 3:**
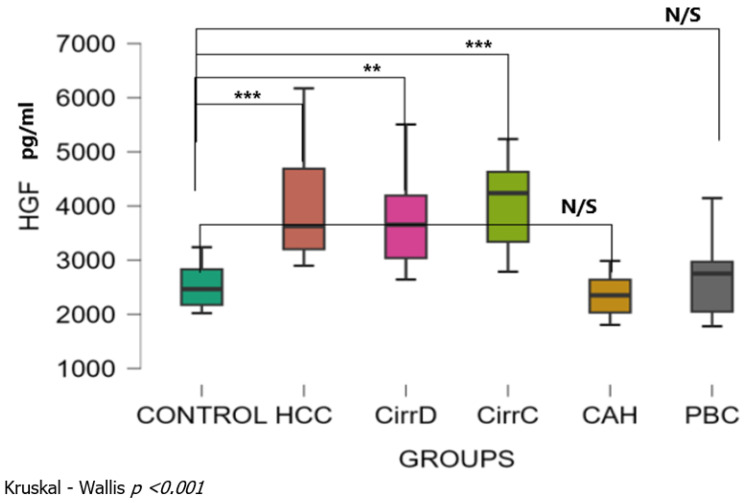
Serum concentrations of HGF in the study groups before treatment. CirrD: Decompensated cirrhosis; CirrC: Compensated cirrhosis; CAH: Chronic viral hepatitis C. **: *p* < 0.01, ***: *p* < 0.001.

There was a significant difference among patient groups (Kruskal–Wallis *p* < 0.001). The mean levels of serum HGF were 2530 ± 399 SD pg/mL for the control group. Levels were significantly increased with a large effect size in HCC patients (3990 ± 1026, *p* < 0.001, r_rb = 0.91), and in patients with decompensated or compensated cirrhosis (3731 ± 834, *p* < 0.01, r_rb = 0.83) and (4057 ± 823, *p* < 0.001, r_rb = 0.89, respectively). Values were not different between chronic hepatitis (2358 ± 374, *p* = 0.44, r_rb = 0.24) and in patients with late stages PBC (2705 ± 718, *p* = 0.57, r_rb = 0.08) compared to controls (Figure 3). Dunn’s post hoc test for pairwise analysis showed that there was no significant difference between HCC and the cirrhotic groups, but there was significant difference between those groups and either CAH or PBC patients.

#### 3.1.4. SCF

**Figure 4:**
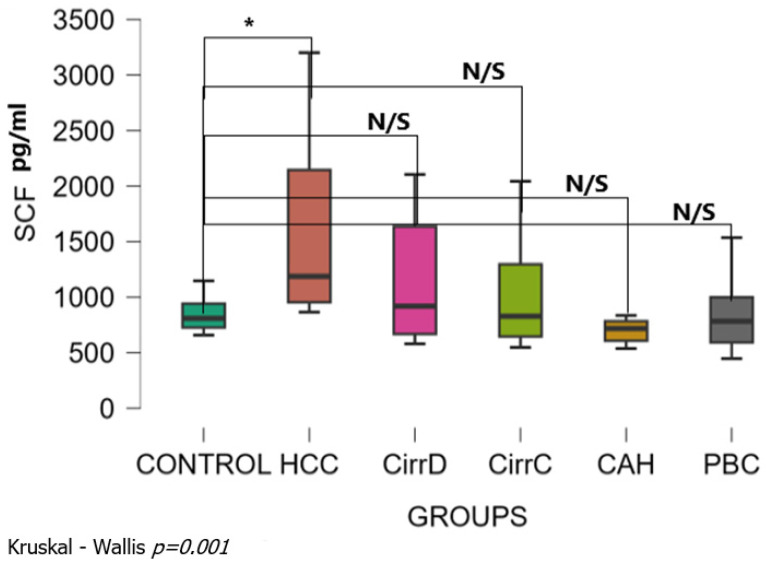
Serum concentrations of SCF in the study groups before treatment. CirrD: Decompensated cirrhosis; CirrC: Compensated cirrhosis; CAH: Chronic viral hepatitis C. *: *p* = 0.001.

Kruskal–Wallis analysis showed that there was a significant difference among patient groups (Kruskal–Wallis *p* = 0.001). The mean levels of serum HGF were 845 ± 153 SD pg/mL for the control group. Levels were significantly increased with a large effect size only in HCC patients (1615 ± 823, *p* < 0.05, r_rb = 0.75). There was no statistical significance between all other groups and the controls: decompensated cirrhosis (1154 ± 551, *p* = 0.37, r_rb = 0.16), compensated cirrhosis (1027 ± 497, *p* = 0.81, r_rb = 0.03), chronic hepatitis (693 ± 102, *p* = 0.67, r_rb = 0.43) and late-stage PBC (845 ± 328, *p* = 0.52, r_rb = 0.11) (Figure 4). Dunn’s post hoc test for pairwise analysis showed that there was a significant difference between HCC and all other groups, but there was no significant difference among those other groups.

#### 3.1.5. VEGF

**Figure 5:**
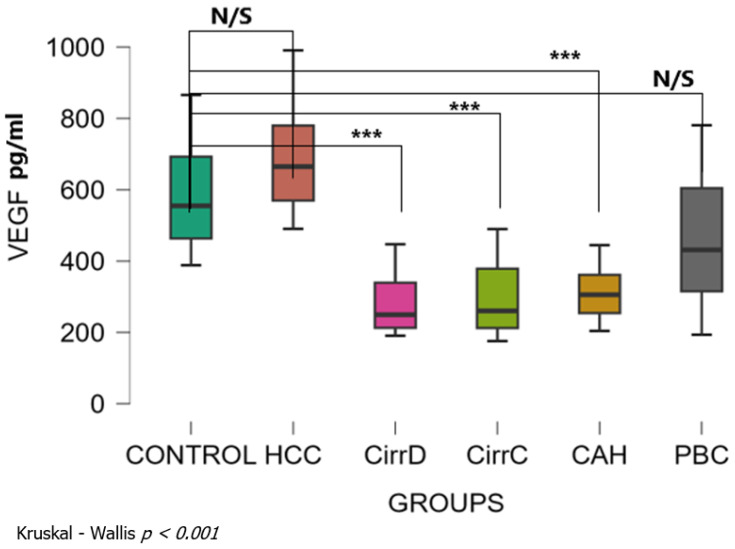
Serum concentrations of VEGF in the study groups before treatment. CirrD: Decompensated cirrhosis; CirrC: Compensated cirrhosis; CAH: Chronic viral hepatitis C. ***: *p* < 0.001.

Kruskal–Wallis analysis showed that there was a significant difference among patient groups (Kruskal–Wallis *p* < 0.001). The mean levels of serum VEGF were 585 ± 153 SD pg/mL for the control group. Levels were not significantly different in HCC patients (690 ± 151, *p* = 0.29, r_rb = 0.38) and in PBC patients (459 ± 179, *p* = 0.059, r_rb = 0.4). There was a statistically significant decrease in the other groups with large effect size: decompensated cirrhosis (280 ± 83, *p* < 0.001, r_rb = 0.95), compensated cirrhosis (297 ± 103, *p* < 0.001, r_rb = 0.89), chronic hepatitis (311 ± 72, *p* < 0.001, r_rb = 0.94) (Figure 5). Dunn’s post hoc test for pairwise analysis showed that there was a significant difference between HCC and all other groups but there was no significant difference among those other groups.

A detailed Dunn’s post hoc analysis between groups is presented in Table 2.

### 3.2. Paired Analysis

The initial values before treatment can be found in Section 3.1 of the results.

#### 3.2.1. Gastrin I

**Figure 6:**
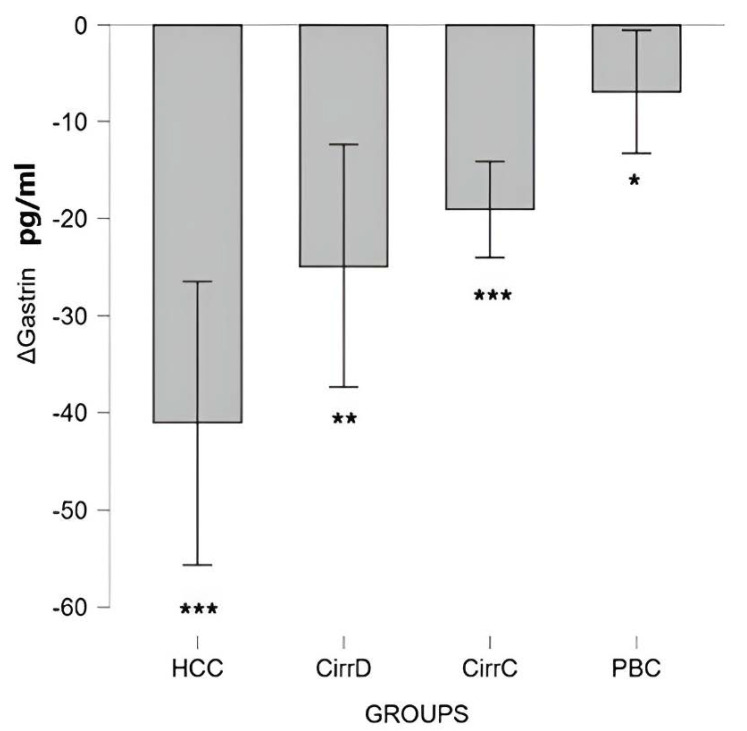
The effect of somatostatin and UDCA administration on the serum levels of Gastrin. Bars represent the difference (Δ) between pre-treatment and post-treatment concentrations plus means and standard deviations. CirrD: Decompensated cirrhosis; CirrC: Compensated cirrhosis; CAH: Chronic viral hepatitis C. *: *p* < 0.05, **: *p* < 0.01, ***: *p* < 0.001.

Results are expressed as difference (Δ) in values before and after treatment and are shown in Figure 6.

HCC: Octreotide administration was associated with a reduction in Gastrin levels to 138 ± 45 pg/mL. Post-treatment Gastrin levels were significantly lower compared to baseline (Wilcoxon signed-rank test: Z = −3.59, *p* < 0.001), indicating a strong treatment effect (r = 0.85).

Decompensated cirrhosis: Somatostatin administration reduced the initial value down to 143 ± 25. Gastrin levels were significantly reduced compared with baseline (Wilcoxon signed-rank test: Z = −3.20, *p* = 0.001). The magnitude of the treatment effect was large (r = 0.75), indicating a strong post-treatment reduction in Gastrin levels.

Compensated cirrhosis: The initial value went down to 128 ± 21. Post-treatment Gastrin levels were significantly lower compared to baseline (Wilcoxon signed-rank test: Z = −3.63, *p* < 0.001), indicating a strong treatment effect (r = 0.85).

PBC: The initial value went down to 164 ± 45 after UDCA administration, *p* = 0.036, mean difference = 6.91 (95% CI 0.53–13.30), with moderate effect size (Cohen’s d_z = 0.54).

#### 3.2.2. IGF-1

HCC: Octreotide administration reduced the values down to 106 ± 26. Post-treatment levels were significantly lower compared to baseline (Wilcoxon signed-rank test: Z = −3.72, *p* < 0.001), indicating a strong treatment effect (r = 0.87).

Decompensated cirrhosis: Somatostatin administration reduced the initial value down to 91 ± 14, *p* < 0.001, mean difference = 7.4 (95% CI 6.4–8.5), with very large effect size (Cohen’s d_z = 3.52).

Compensated cirrhosis: The initial value went down to 94 ± 19. Post-treatment levels were significantly lower compared to baseline (Wilcoxon signed-rank test: Z = −3.72, *p* < 0.001), indicating a strong treatment effect (r = 0.87).

**Figure 7:**
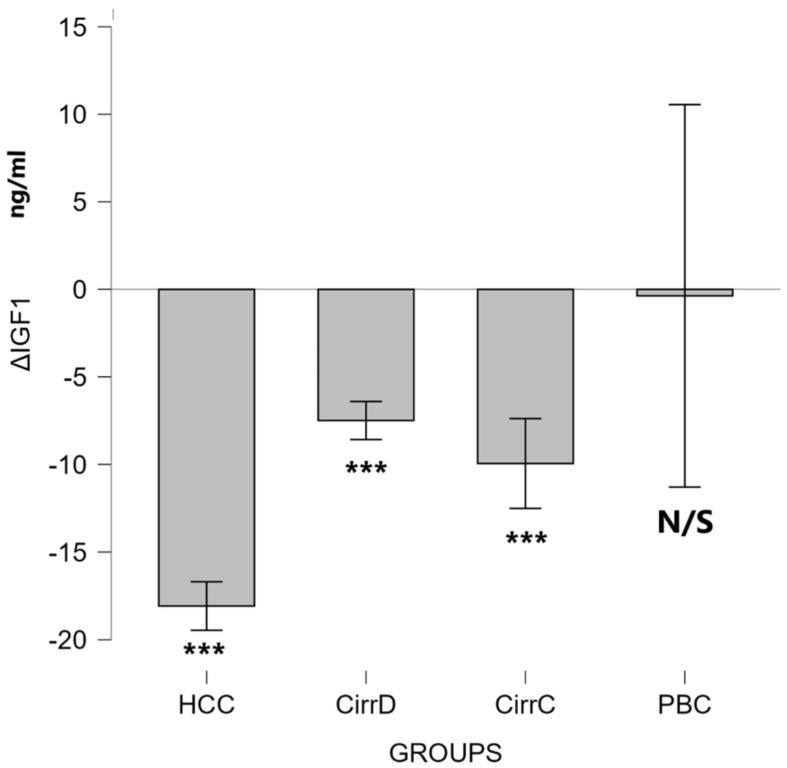
The effect of somatostatin and UDCA administration on the serum levels of IGF1. Bars represent the difference (Δ) between pre-treatment and post-treatment concentrations plus means and standard deviations. CirrD: Decompensated cirrhosis; CirrC: Compensated cirrhosis; CAH: Chronic viral hepatitis C. ***: *p* < 0.001.

PBC: After UDCA administration there was no change in the serum value (164 ± 28, *p* = 0.944). Results are shown in Figure 7.

#### 3.2.3. HGF

HCC: Octreotide administration increased the values up to 4541 ± 1704. Post-treatment levels were significantly lower compared to baseline (Wilcoxon signed-rank test: Z = −2.67, *p* < 0.001), indicating a moderate treatment effect (r = 0.62).

Decompensated cirrhosis: Somatostatin administration increased the initial value up to 4122 ± 816, *p* < 0.001, mean difference = 391.3 (95% CI 297–485), with very large effect size (Cohen’s d_z = 2.06).

Compensated cirrhosis: The initial value went up to 4307 ± 870, *p* < 0.001, mean difference = 250.4 (95% CI 167.6–333.2), with very large effect size (Cohen’s d_z = 1.5).

**Figure 8:**
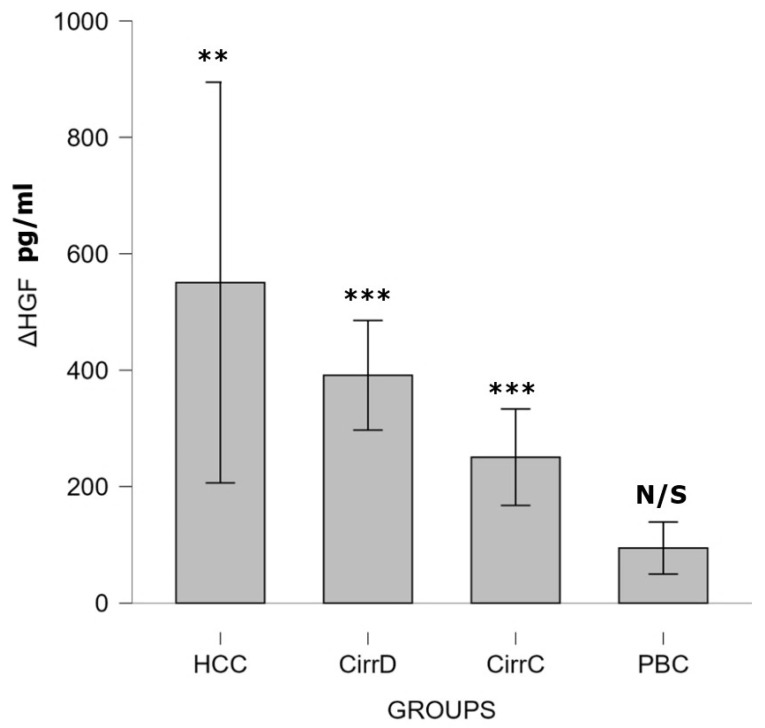
The effect of somatostatin and UDCA administration on the serum levels of HGF. Bars represent the difference (Δ) between pre-treatment and post-treatment concentrations plus means and standard deviations. CirrD: Decompensated cirrhosis; CirrC: Compensated cirrhosis; CAH: Chronic viral hepatitis C. **: *p* < 0.01, ***: *p* < 0.001.

PBC: After UDCA administration, it had no significant effect compared to the pre-treatment level (2799 ± 675, *p* = 0.78). Results are shown in Figure 8.

#### 3.2.4. SCF

HCC: Octreotide administration decreased the values down to 1004 ± 202. Post-treatment levels were significantly lower compared to baseline (Wilcoxon signed-rank test: Z = −3.72, *p* < 0.001), indicating a strong treatment effect (r = 0.87).

**Figure 9:**
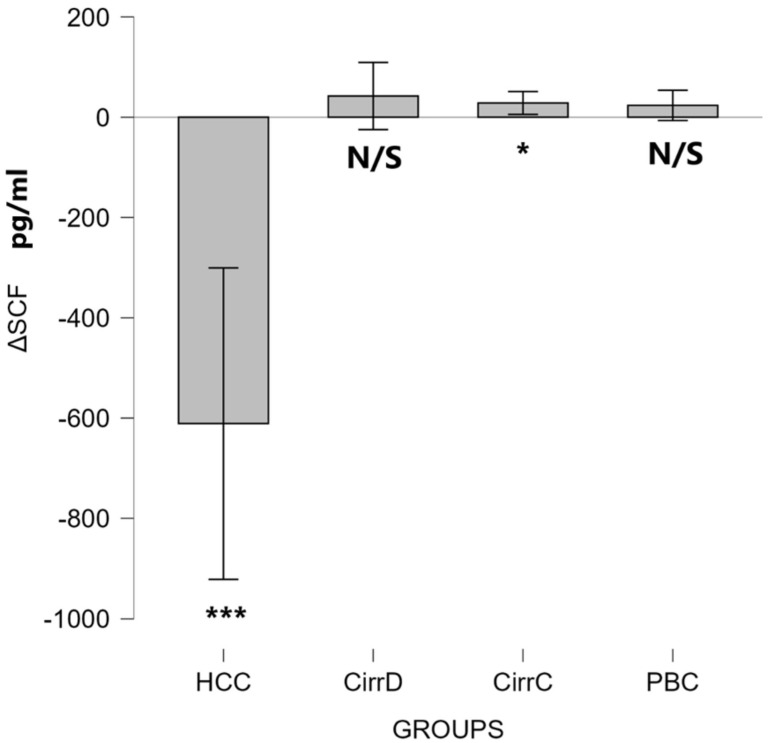
The effect of somatostatin and UDCA administration on the serum levels of HGF. Bars represent the difference (Δ) between pre-treatment and post-treatment concentrations plus means and standard deviations. CirrD: Decompensated cirrhosis; CirrC: Compensated cirrhosis; CAH: Chronic viral hepatitis C. *: *p* < 0.05, ***: *p* < 0.001.

As can be seen in Figure 9, the treatment had virtually no effect on the other patient groups.

#### 3.2.5. VEGF

HCC: Octreotide administration decreased the values down to 520 ± 231, *p* < 0.001, mean difference = 169.3 (95% CI 128.4–210.3), with very large effect size (Cohen’s d_z = 2.05).

Decompensated cirrhosis: There was no change in the post-treatment value (274 ± 58, *p* = 0.586). On the contrary, in patients with compensated cirrhosis, the initial value was significantly reduced to 215 ± 47, *p* < 0.001, mean difference = 81.5 (95% CI 53.5–109.5), with very large effect size (Cohen’s d_z = 1.44).

**Figure 10:**
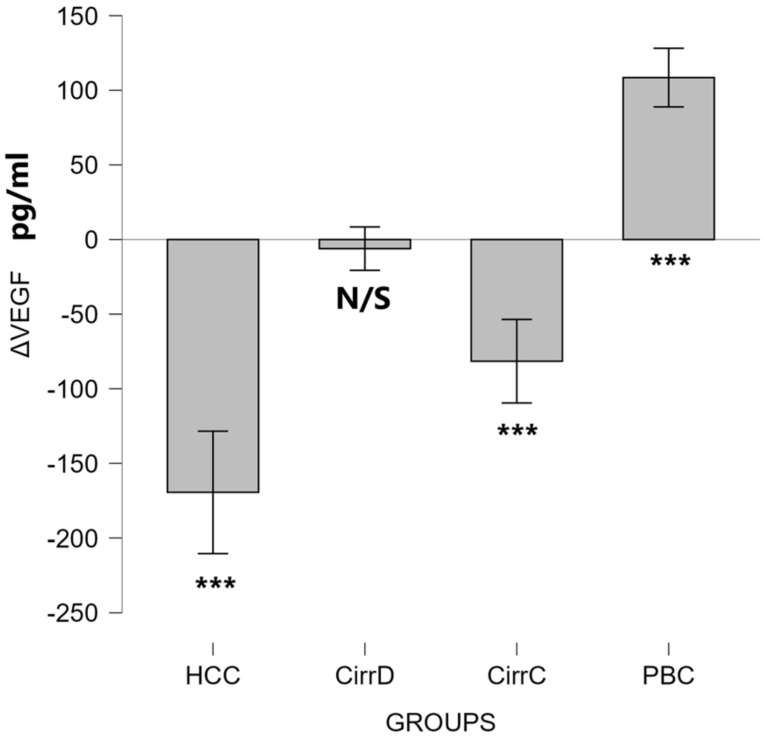
The effect of somatostatin and UDCA administration on the serum levels of HGF. Bars represent the difference (Δ) between pre-treatment and post-treatment concentrations plus means and standard deviations. CirrD: Decompensated cirrhosis; CirrC: Compensated cirrhosis; CAH: Chronic viral hepatitis C. ***: *p* < 0.001.

PBC: After UDCA administration, VEGF values were significantly increased (568 ± 214, *p* < 0.001), mean difference = 108.5 (95% CI 88.8–128.1), with very large effect size (Cohen’s d_z = 2.74). Results are shown in Figure 10.

Detailed Dunn’s post hoc analysis of Δ means between groups are presented in Table 3.

## 4. Discussion

Somatostatin has been implicated in the treatment of HCC, as there is extensive experimental evidence both in vitro and in vivo of its anti-tumoral effects [31]. Clinical trials have also suggested that octreotide may be effective in selected patients [37]. In addition to several anti-tumoral mechanisms, somatostatin and its analogs repress several trophic factors implicated in HCC progression, such as IGF1 and IGF2 [12]. Other potential tumor trophic factors such as Gastrin, glucagon and insulin are also inhibited by somatostatin, but their exact role in HCC has not been fully clarified [38]. HCC-associated angiogenesis is also inhibited by octreotide [39], either through direct binding to the endothelium or indirectly through the inhibition of the vascular endothelial growth factor (VEGF) [40].

However, information on the effects of somatostatin on trophic factors under clinical conditions is very limited. Therefore, we studied the basic levels in patients with different stages of chronic HCV and compared them to normal controls. Moreover, the effects of somatostatin in five trophic factors before and after treatment with either somatostatin or its analog octreotide were studied in patients with HCV-related HCC or cirrhosis. PBC patients were included in order to identify the basic levels of the growth factors in a model of chronic fibrotic cholestatic disease and see if treatment with a mechanistically different drug would have an impact on these levels.

It should be noted that the results were similar between somatostatin and octreotide, with the exception of SCF. This finding requires further investigation, since there is no evidence so far that somatostatin and its analog octreotide differ in their anti-neoplastic and anti-inflammatory effects, despite the fact that octreotide only binds to somatostatin receptors 2.5 and, to a lesser degree, to receptor 3 [31,36].

Due to the exploratory and retrospective design, multiple biomarkers and subgroup analyses were performed, which may increase the risk of Type I error. To mitigate this, post hoc corrections were applied where appropriate. Conversely, the relatively small sample size in specific subgroups, such as the HCC cohort, may limit statistical power and increase the risk of Type II error. Therefore, non-significant findings should be interpreted with caution, and effect sizes were reported to aid interpretation beyond *p*-values.

### 4.1. Gastrin

Serum Gastrin levels were significantly increased in all study groups, including PBC compared to controls. Gastrin and progastrin are synthesized in the stomach and metabolized in the liver, but their levels in various hepatic disorders have not been adequately studied. Patients with chronic viral hepatitis and cirrhosis had significantly higher plasma levels of progastrin and Gastrin, in agreement with our study [41].

There is sufficient evidence to indicate that Gastrin is involved in HCC. The Gastrin receptor cholecystokinin-B receptor (CCK-BR) is overexpressed in HCC liver and HepG2 liver cancer cells, but is absent from normal liver tissue [42]. Gastrin stimulation of HepG2 cells induced their proliferation, which is blocked by the CCK-BR antagonist proglumide [43]. Moreover, proglumide increased the number of intra-tumoral CD8+ cytotoxic T cells in experimental HCC. Proglumide, in combination with an antibody against PD-1, further significantly increased CD8+ T cells, and animal survival was improved [5]. Progastrin, an 80 amino acid protein, is the precursor of Gastrin. In an HCC cohort, median hPG80 significantly decreased from 11.54 pM at inclusion down to 1.99 pM in remission. [44,45]. Whether the reduction in Gastrin levels we found is also associated with disease remission cannot be answered by our study and requires further research. Interestingly, we found a significant reduction in the increased levels of Gastrin in PBC patients treated with UDCA. Whether this is protective for liver and stomach tumorigenesis remains to be investigated. In the stomach, Gastrin may have different effects, as only progastrin promoted antral carcinogenesis, while amidated Gastrin (G-17) inhibited gastric cancer initiation [46]. Gastrin-deficient mice developed spontaneous antral tumors [47] while hypergastrinemic mice are resistant to antral carcinogenesis [48], probably because of the inhibition of stem cells in the antrum [3].

### 4.2. IGF 1

We found significantly decreased levels of IGF-1 in all study groups. Moreover, somatostatin administration further reduced IGF-1 in HCC and all cirrhotic patients. UDCA had no effect on patients with PBC.

Most results in the literature are in agreement with our findings. HCC associated with viral cirrhosis had significantly lower levels of serum IGF1 compared to controls [49,50,51,52,53,54], probably due to reduced IGF1 synthesis by damaged hepatocytes [52]. IGF-1 levels could be considered as a surrogate marker for the hepatic reserve [55,56]. However, an association was demonstrated between decreased serum IGF-1 and the development of HCC, which was independent of the grade of hepatic dysfunction [57]. The reduction in IGF1 was greater for the virus-associated HCC compared to non-infected HCC patients [51]. Patients with HCV-related HCC had lower levels of IGF1 than those with HBV-related HCC [58]. We selected only patients with HCV-related disease to avoid possible interference in the results by different etiological factors.

Low serum levels of IGF1 significantly correlated with poor overall survival in patients with HCC [59]. This was also reported even in patients after arterial chemoembolization (TACE) [60,61]. IGF1 levels were also an indicator of recurrence after treatment. Preoperative and postoperative serum levels of IGF-1 and IGFBP-3 were tightly associated with the recurrence of HCC [62,63]. Delayed recovery of IGF-1 level at 30 days after liver resection was an independent risk factor for early recurrence in HCC patients [64].

A lower serum level of IGF-1 was associated with more advanced stages of cirrhosis, which was not found in our study (Table 2), as there was no difference between decompensated and compensated cirrhosis [65,66]. IGF-1 levels were also significantly lower in patients with cirrhosis or sarcopenia in patients with HCC treated with stereotactic body radiotherapy [67]. A systematic review of 20 studies finally concluded that low serum IGF1 levels were predictive of shorter time-to-progression and reduced overall survival in HCC patients [68].

However, there is some controversy about the role of IGF1. Some studies have demonstrated that high serum IGF1 and low serum IGF-binding protein 3 (IGFBP3) are associated with an increased risk of several cancers including HCC [69]. Therefore, it has been suggested that it is the IGF1/IGFBP3 ratio, more than IGF1 itself, which is involved in hepatocarcinogenesis. In addition, IGF-1R was highly expressed in the HCC cell lines promoting cell proliferation and anti-apoptosis, resulting in HCC resistance to sorafenib [70]. Moreover, it has been suggested that IGF1 has a positive effect on HCC growth through the inhibition of cathepsin B (CTSB) degradation. In HCC cell lines, IGF1 did not change the CTSB mRNA levels, but prolonged the half-life of cathepsin B. Therefore, reduced IGF1 levels might be interpreted as a compensatory mechanism against tumor growth [71]. In that respect, the significant reduction that we observe after octreotide administration may be beneficial. Similar results to our study have been reported in an earlier report. IGF-1 was significantly reduced after 3 mo of treatment with octreotide in HCC Child A and B patients [72]. The role of IGF-1 in HCC has been extensively reviewed [73].

### 4.3. HGF

We found significantly increased levels in our study groups, with the exception of chronic hepatitis and PBC. HGF plays an important role in HCC progression. In previous clinical studies, the serum HGF level correlated positively with the tumor metastasis of HCC. Moreover, expression of c-Met, the receptor tyrosine kinase of HGF, was closely associated with early recurrence [74,75]. HGF can be secreted from cancer-associated fibroblasts in the tumor microenvironment for the initiation of cirrhosis-associated HCC [76,77]. The aberrant activation of HGF/c-Met axis is implicated in several aspects of HCC development, including promoting tumor initiation, proliferation, invasion and even inducing drug resistance [22,78].

Several studies are in agreement with our results. In an earlier study, liver tumor patients had significantly higher serum levels of HGF compared to controls. In addition, significantly higher levels of HGF were found in serum from HCC patients with metastasis compared to HCC patients without metastasis [79]. Also, the serum concentrations of HGF were significantly higher in patients with HCV related HCC compared to patients with CH or cirrhosis [80,81]. This was true in our study only for chronic hepatitis, where levels were similar to controls and significantly lower than HCC. All patients with cirrhosis had similarly increased levels with HCC. These results might indicate that somatostatin and octreotide may indeed favor the progression of HCC.

However, HGF/c-MET axis is also implicated in liver regeneration [82]. Hence, the increase after somatostatin may be beneficial. This requires further investigation.

### 4.4. SCF

Only a small increase was found in the SCF serum levels in HCC. This might be due to the production of SCF by oval cells, as these cells work throughout the development of HCC [26]. No significant differences were found in the other groups. SCF is mostly involved in liver regeneration, which is assisted by the synergistic interaction of GM-CSF and SCF, and is made possible via pathways such as TGF-β signaling [83]. Increased serum levels were found in a murine model of acetaminophen-induced liver toxicity correlating with liver regeneration [84]. In that respect, it was demonstrated that the SCF level was significantly increased in patients with chronic HCV hepatitis compared to normal controls, suggesting that SCF could contribute to liver repair. Significantly higher serum levels of SCF in HCV patients were also shown after the achievement of SVR when compared to healthy individuals [2,85]. These results could not be verified in our study. Moreover, there was no effect of either somatostatin or UDCA on the serum levels of SCF, in contrast to octreotide that caused a significant reduction in HCC patients. This may be alarming, as SCF supports the differentiation of CD34+ human hematopoietic cells into natural killer (NK) cells in the presence of IL-2 to help the immune response to tumors [86]. On the other hand, this reduction may be beneficial, indicating a suppression of oval cells in HCC [26].

### 4.5. VEGF

In our study, a slight but non-significant increase was observed in HCC. However, a significant reduction was found in all patients with cirrhosis, while PBC had levels similar to controls.

Among the multitude of pro-angiogenic factors, the most potent is VEGF, which is secreted primarily by cancer cells in the liver and has the highest specificity for endothelial cells. It regulates the formation of tumor blood vessels, promoting HCC growth by binding to its receptors on the membrane of endothelial cells [87,88]. Several studies have shown that VEGF is frequently expressed in HCC [89,90,91], but only in 60% of HCC without metastasis and over 90% with metastasis. The abnormal expression levels of VEGF in sera of HCC patients were directly correlated with the metastasis and recurrence of tumors [92]. This might explain the non-significant increase in patients with HCC in our study, as patients with metastases were not included.

Some studies have reported different results from our study. Serum VEGF level in the HCC patients was significantly higher compared to healthy controls. Interestingly, serum VEGF levels were significantly correlated with platelet counts. A previous study from the same group found that increased VEGF levels were also significantly associated with venous invasion and advanced tumor stage [93,94]. PLTs are possibly the reason for the discrepancy. Indeed, platelets are considered to be the major source of VEGF in the human body [95,96].

Another study confirmed the relation between elevated serum VEGF in HCC patients and tumor size. This study also showed that significantly higher serum VEGF levels were found in patients with macroscopically evident portal vein invasion and metastasis [97]. High levels of VEGF after TACE were reported. Responders to TACE had lower levels of VEGF than non-responders both before and after TACE. However, VEGF was not associated with overall survival [98], contrary to the meta-analysis in patients treated with sorafenib, where high levels of VEGF were associated with poor overall and poor progression-free survival in HCC [99].

It has been reported that liver-specific VEGF depletion led to fenestration loss and HSCs activation, promoting fibrosis. Reduced VEGF therefore promotes fibrosis and indirectly promotes HCC and is consistent with our findings. However, in advanced fibrosis VEGF exhibits paradoxical pro-fibrotic effects [100]. Therefore, the reduced levels of VEGF we found after treatment with somatostatin or octreotide may be consistent with a reduction in fibrosis, since our patients had advanced fibrosis.

In terms of response to octreotide, there was a discrepancy between our results and an earlier report. Contrary to our findings, VEGF-A was not significantly reduced after 3 months of treatment with octreotide in HCC Child A and B patients. The significant reduction we found might be explained by the fact that our measurements were performed after 6 months of treatment [72]. An alternative explanation would be that platelets may influence VEGF levels, and data on the number of platelets or their activation were not included in the calculation of our results. This is certainly a limitation of the study. A similar reduction to ours was reported in a study of the anti-VEGF antibody, bevacizumab, in advanced HCC. Plasma VEGF levels decreased from baseline in all patients after 8 weeks of bevacizumab therapy [101]. In another report, VEGF levels were increased after treatment of HCC. However, the treatment regime did not include octreotide [81].

There is another point that could benefit from VEGF reduction. VEGF is a well-documented survival factor that inhibits the apoptosis of both endothelial and epithelial cells. Therefore, a reduction by somatostatin may increase apoptosis in HCC [102,103]. On the other hand, reduced angiogenesis increases tumor hypoxia, leading to increased expression of HIF-1a, contributing to the resistance to anti-angiogenesis therapy in hepatocellular carcinoma (HCC) [104,105].

There are some inherent limitations of the study. The retrospective analysis, the relatively small sample size in certain subgroups, and the absence of longitudinal validation are definite drawbacks of this study, and a larger study is required in the future.

In conclusion, the findings of the present retrospective observational study indicate that somatostatin or its analog octreotide have the same effect on growth factors that may be implicated in hepatocellular carcinoma, with the exception of SCF that is reduced by octreotide. This may be either detrimental or beneficial and further studies are required to identify the balance between benefit and damage during modulation of trophic factors. Nonetheless, this is not a treatment trial to demand the meticulous matching of patients in terms of inflammation and liver reserve. However, octreotide administration has effects that justify its use as an adjunct treatment of HCC in future trials. Interestingly, UDCA administration in PBC had an unexpected effect besides the expected reduction in Gastrin. The significant increase in serum VEGF requires further investigation.

## Figures and Tables

**Table 1:** Demographics of patients.

	HCC	Decomp Cirrhosis	Compensated Cirrhosis	Chronic Hepatitis	PBC	Controls
No	19	19	18	19	18	17
Age range	45–68	41–70	37–59	36–60	35–63	40–60
Sex (male/female)	14/5	12/7	15/3	13/6	3/15	12/5
Esophageal varices	19	17	5	0	9	
Child–Pugh score (A/B/C)	0/19/0	0/10/9	0/18/0	5/14/0	6/12/0	
MELD (mean ± SD)	17 ± 3	25 ± 4	16 ± 5	10 ± 2	13 ± 3	
Etiology	HCV	HCV	HCV	HCV		
Stage (III/IV)					5/13	

**Table 2:** Pairwise Dunn’s post hoc comparisons. ×: No statistical significance (*p*-value > 0.05).

Groups	Gastrin	IGF1	HGF	SCF	VEGF
HCC–CirrD	×	×	×	×	*p* < 0.001
HCC–CirrC	×	×	×	×	*p* < 0.001
HCC–CAH	*p* < 0.001	*p* = 0.009	*p* < 0.001	*p* < 0.001	*p* < 0.001
HCC–PBC	×	×	*p* = 0.002	*p* = 0.002	×
CirrD–CirrC	×	×	×	×	×
CirrD–CAH	*p* < 0.001	*p* < 0.001	*p* < 0.001	×	×
CirrD–PBC	×	*p* < 0.001	*p* = 0.013	×	*p* = 0.036
CirrC–CAH	*p* = 0.009	*p* < 0.001	*p* < 0.001	×	×
CirrC–PBC	×	*p* = 0.006	*p* < 0.001	×	×
CAH–PBC	*p* < 0.001	×	×	×	×

**Table 3:** Pairwise Dunn’s post hoc comparisons of Δ means before and after drug treatment. ×: No statistical significance (*p*-value > 0.05).

Groups	Gastrin	IGF1	HGF	SCF	VEGF
HCC–CirrD	×	*p* < 0.001	×	*p* < 0.001	*p* < 0.001
HCC–CirrC	×	*p* < 0.001	×	*p* < 0.001	×
HCC–PBC	*p* < 0.001	*p* = 0.02	*p* = 0.04	*p* < 0.001	*p* < 0.001
CirrD–CirrC	×	×	×	×	×
CirrD–PBC	×	×	*p* = 0.03	×	*p* = 0.026
CirrC–PBC	×	×	*p* = 0.03	×	*p* < 0.001

## Data Availability

The raw data supporting the conclusions of this article will be made available by the authors on request.
